# Effects of colostrum serum on the serotonergic system in the dorsal raphe nuclei of exercised rats

**DOI:** 10.20463/jenb.2017.0047

**Published:** 2017-03-31

**Authors:** Tae-Woon Kim, Chang-Ju Kim, Jinhee Seo

**Affiliations:** 1 Department of Physiology, College of Medicine, KyungHee University, Seoul Republic of Korea; 2 Division of Adaptive Physical Education, Baek Seok University, Cheonan Republic of Korea

**Keywords:** Colostrum serum, Central fatigue, Serotonergic system, Exhaustive exercise

## Abstract

**[Purpose]:**

The central fatigue hypothesis suggests that exhaustion, or the maximum level of exercise, induces excessive stress and increases serotonin concentrations in the brain, which in turn decreases central nervous system (CNS) function and induces fatigue. Our aim was to determine the effects of colostrum serum on the serotonergic system in the dorsal raphe nuclei during exhaustive exercise.

**[Methods]:**

Animals were randomly divided into five groups: control, exercise, exercise and treatment with 50, 100, and 200 mg/kg of colostrum serum. The rats in the colostrum serum treatment groups were fed colostrum serum at three different doses of 50, 100, and 200 mg/kg per day for seven days. The rats in the control and exercise groups received water by oral gavage once per day for seven days.

**[Results]:**

The time to exhaustion in response to treadmill running increased after treatment with colostrum serum. These results show that exhaustive exercise led to over activation of the serotonergic system in the dorsal raphe nuclei, and that treatment with colostrum serum suppressed of the exercise-induced expression of tryptophan hydroxylase (TPH) and serotonin (5-HT). The results also indicated that exhaustive exercise induced 5-HT_1A_ autoreceptor and serotonin transporter (5-HTT) overexpression in the dorsal raphe nuclei, and that colostrum serum treatment suppressed exhaustive exercise-induced 5-HT_1A_ and 5-HTT expression in the dorsal raphe nuclei. The most effective dose of colostrum serum was 100 mg/kg.

**[Conclusion]:**

Overall, our study suggests that colostrum serum has positive effects on exercise performance and recovery by increasing the resistance to fatigue.

## INTRODUCTION

The activity in the brain that mediates the desire for optimal exercise performance is influenced by many factors. Such activity affects the cross-bridges of the muscles, which drive muscle contraction. The mechanisms involved in fatigue during exercise are also initiated in the central nervous system (CNS). The causes of the fatigue that occurs during exercise include psychological factors, health conditions, environmental factors, depletion of the energy sources required for muscle activities, the accumulation of metabolites that are produced during exercise, and disruption of the enzyme systems involved in energy metabolism^1^. Very intense endurance exercise training is associated with symptoms such as a decrease in exercise performance, fatigue, mood disorders, somnipathy, loss of appetite, and anxiety^2^. The fatigue-related reduction in skeletal muscle power is caused by alterations in efferents from the brain, since the brain does not recruit additional motor units during prolonged exercise because additional recruitment would threaten the capacity to maintain homeostasis, damage various organs, and reduce exercise performance^3^. The situation predicts that an increased perception of discomfort is produced by exhaustive exercise^4^. Serotonin (5-hydroxytryptamine or 5-HT), which can act as a cerebral vasoconstrictor, is associated with depression, sleep, and mood disorders. There is growing interest in the role of serotonin as a key factor in the induction of fatigue. Notably, fluctuations in serotoninergic activity during prolonged exercise induce and delay fatigue^5-7^. Regular and adequate exercise can improve the mood of patients with depression, while excessive exercise may induce mood disorders. 

The central fatigue hypothesis suggests that exercising to exhaustion induces excessive stress on the CNS, which then induces an excess of serotonin synthesis and metabolism in the brain leading to fatigue and a reduction in exercise performance in both humans and animals^8-10^. Serotonin is mainly synthesized in serotonergic neurons in the raphe nuclei of the midbrain and in neurosecretory cells in the pineal gland. Tryptophan hydroxylase (TPH) is an important enzyme that is involved in the synthesis of serotonin^11^. The modulation of the TPH gene is crucial for the regulation of the serotonergic system. An increase in TPH mRNA levels enhances TPH activity and the effects of serotonin^12^. 

Sports drinks have recently been developed to improve endurance exercise abilities, athletic performance, and recovery from fatigue by enhancing muscle strength^13^ and suppressing muscle fatigue^14^. Similar to sports drinks, milk has recently been recognized to be highly effective for rehydration after resistance or endurance exercises^15^, with some studies showing that milk intake after exercise is as effective as drinks containing both carbohydrates and electrolytes^16^. Colostrum, which is secreted from a mother during the first 72 h after infant delivery, is particularly abundant in essential nutrients and bioactive factors, including cytokines and various growth factors that promote cell proliferation, collagen and hyaluronic acid production, epithelial cell growth, and angiogenesis^17^. These molecules play an important role in immunity, growth, and biodefense functions, such as wound healing and antibiotic functions^18, 19^. Thus, colostrum is an ample source of energy and nutrients for newborns. It also aids in the defense against pathogenic microorganisms and immature intestinal development, and in the support of organ and tissue growth and immune system development^20, 21^. The present study examines the ergogenic effects of colostrum. Specifically, we investigate whether colostrum delays the central fatigue that is induced by high-intensity exhaustive exercise through activation of the serotonergic system in the dorsal raphe nuclei of the rat midbrain. 

## METHODS

### Animals Treatments

Male Sprague-Dawley rats weighing 150.6 ± 10 g (six weeks old) were obtained from a commercial breeder (Orient Co., Seoul, Korea). The experimental procedures were performed in accordance with the animal care guidelines of the National Institutes of Health (NIH) and the Korean Academy of Medical Sciences. The rats were housed under controlled temperature (20 ± 2°C) and lighting (07:00 to 19:00 h) conditions with food and water available *ad libitum*. Animals were randomly divided into five groups: control, exercise, exercise and 50 mg/kg of colostrum serum, exercise and 100 mg/kg of colostrum serum, and exercise and 200 mg/kg of colostrum serum (n=10 per group). The colostrum serum solution was diluted in the drinking water at concentrations of 50, 100, and 200 mg/kg, and these doses were made up fresh daily. The rats in the colostrum serum treatment groups received colostrum serum at three different doses (50, 100, and 200 mg/kg of body weight, respectively) each day for seven days. The colostrum serum was administered 60 min prior to the start of exercise, which was conducted as described below. The rats in the control and exercise groups received water by oral gavage once per day for seven days. 

### Measurement of exercise abilities

To measure the effectiveness of colostrum serum in improving exercise ability, all-out running time was measured on a treadmill (Exer-6M treadmill, Columbus instruments, USA) and used as an index for exercise performance. For treadmill adaptation training, an exercise load of 30 min per day for three days was performed. The 30 min treadmill exercise began at a speed of 10 m/min for the first 10 min, increased to 16 m/min for the next 10 min, and increased further to 21 m/min for the last 10 min. Exhaustion was assessed following the conclusion of the experiment on day seven by making the animals run on a treadmill at a speed of 10 m/min for the first 5 min, after which time the treadmill speed was incrementally increased to 16, 18, 21, 24, 26, 29, 32, 34, and 37 m/min with 5 min intervals. The treadmill speed was then increased to 40 m/min until the animals were exhausted^6, 22^. The animals were considered to be exhausted at the point when they could no longer maintain their balance running at speed for 3 min. In order to avoid the effects of electric shock stress, we used a sponge as a tactile incentive rather than an electric shock-plate. 

### Tissue preparation

The animals were sacrificed on the seventh day of the experiment, immediately following the determination of the exhaustion time point. To prepare the brain for sectioning, the animals were fully anesthetized with Zoletil 50^®^ (10 mg/kg, intraperitoneally [i.p.; Vibac, Carros, France), following which the rats were transcardially perfused with 50 mM phosphate-buffered saline (PBS) and then fixed with a freshly prepared solution of 4% paraformaldehyde in 100 mM phosphate buffer (PB; pH 7.4). The brains were then removed from the skulls, postfixed in the same fixative overnight, and transferred into a 30% sucrose solution for cryoprotection. Coronal sections with thicknesses of 40 μm were cut using a freezing microtome (Leica, Nussloch, Germany). 

### Immunohistochemistry for TPH and 5-HT

To visualize TPH and 5-HT expression, immunohistochemistry for TPH and 5-HT was performed in the dorsal raphe nuclei. The dorsal raphe nuclei, spanning from Bregma -7.20 mm to -8.00 mm, were obtained from each brain. The sections were incubated in PBS for 10 min, and then washed three times in the same buffer. The sections were then incubated in 1% hydrogen peroxide (H2O2) for 30 min. The sections were incubated overnight with a rabbit anti-TPH antibody (1:1000; Oncogene Research Products, Cambridge, UK) and a rabbit anti-5-HT antibody (1:500, Abcam, Cambridge, UK). The sections were subsequently incubated with a biotinylated rabbit secondary antibody (1:200; Vector Laboratories) for another 1 h. The signal was amplified with the Vector Elite ABC kit^®^ (1:100; Vector Laboratories). Antibody-biotin-avidin-peroxidase complexes were visualized using 0.03% DAB, and the sections were mounted onto gelatin-coated slides. The slides were air-dried overnight at room temperature, and the coverslips were mounted using Permount^®^. 

### Western blot for 5-HT_1A_ and 5-HTT

The dorsal raphe nuclei were collected and immediately frozen at -70°C. Proteins were extracted from the dorsal raphe nuclei samples. The tissues were homogenized with a lysis buffer containing 50 mM Tris–HCl (pH 8.0), 150 mM NaCl, 10% glycerol, 1% Triton X-100, 1.5 mM MgCl_2_·6H_2_O, 1 mM EGTA, 1 mM PMSF, 1 mM Na_2_VO_4_, and 100 mM NaF, and then centrifuged using an ultra-centrifuge at 50,000 rpm for 1 h. The protein content in the samples was measured using a Bio-Rad colorimetric protein assay kit (Bio-Rad, Hercules, CA, USA). The samples (30 μg protein) were separated on sodium dodecyl sulfate (SDS)-polyacrylamide gels, and then transferred to nitrocellulose membranes. Anti-β actin (1:1000; Santa Cruz), anti-5-HT_1A_ (1:1000; Abcam), and anti-5-HTT (1:1000; Abcam) were used as the primary antibodies. A horseradish peroxidase-conjugated anti-mouse secondary antibody was used for β-actin, and an anti-rabbit secondary antibody was used for 5-HT_1A_ and 5-HTT. Experiments were performed under normal laboratory conditions and at room temperature, except for the transfer of proteins to membranes. Proteins were transferred to the nitrocellulose membranes at 4ºC using a cold pack and pre-chilled buffer. The detection of bands was performed using the enhanced chemiluminescence (ECL) detection kit (GE healthcare, Amersham^TM^). To compare the relative expression of proteins, densitometry analyses were carried out on the bands detected using Molecular AnalystTM, version 1.4.1 (Bio-Rad). 

### Colostrum serum extraction method

For the production of colostrum serum, bovine colostrum that was milked on the farm and refrigerated for no more than 72 h was centrifuged at 3000 x g for 30 min, resulting in colostrum free from milk fat. To remove any residual substances, colostrum without fat was collected using filter paper. The colostrum that was collected was mixed with 2N HCl to adjust it to a pH of 4.6, corresponding to the isoelectric point of casein, and this was followed by slow stirring for 1 h to precipitate the casein. For complete separation and precipitation, the colostrum was centrifuged at 8700 x g for 30 min and then the supernatant was collected. The filtrate was filtered through a 0.2 μm diameter membrane filter on a clean bench to remove any pathogenic microorganisms, resulting in the final bovine colostrum extract. 

### Data analysis

To confirm the expression of 5-HT_1A_ and 5-HTT proteins, the bands detected were analyzed densitometrically using Molecular AnalystTM software, version 1.4.1. The numbers of TPH and 5-HT positive cells in the dorsal raphe nuclei were counted hemilaterally using a light microscope (Olympus, Tokyo, Japan) and Image-Pro^®^ Plus (Media Cyberbetics Inc.). Values are expressed as the means ± standard error of the means. Data were analyzed using a one-way analysis of variance (ANOVA), followed by a Duncan’s post hoc test using SPSS software (SPSS, Inc., Chicago, IL, USA). Differences with a p-value <0.05 were deemed to be statistically significant. 

## RESULTS

### Effects of colostrum serum on the time to exhaustion during treadmill running

**Figure 1.**
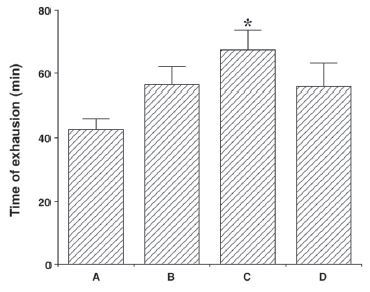
Treadmill running time to exhaustion to treatment with colostrum serum. A: exercise group, B: exercise and 50mg/kg of colostrum serum treatment, C: exercise and 100mg/kg of colostrum serum treatment, D: exercise and 200mg/kg of colostrum serum treatment. The data are presented as the mean ± standard error of the mean (S.E.M). * *p* < 0.05 compared to the exercise group.

The effects of colostrum serum on the time to exhaustion are presented Fig. 1 The average time to fatigue was 42.33 ± 3.40 min for the exercise group, 56.50 ± 5.79 min for the exercise + 50 mg/kg colostrum serum group, 67.44 ± 6.17 min for the exercise + 100 mg/kg colostrum serum group, and 55.87 ± 7.52 min for the exercise + 200 mg/kg colostrum serum group. These findings indicate that the time to exhaustion in response to treadmill running increased following treatment with colostrum serum (F(3,35)=2.99, p=0.047). Specifically, treatment with 100 mg/kg of colostrum serum significantly increased the time to exhaustion induced by treadmill running when compared with the exercise only group (p=0.027). 

### Effects of colostrum serum on TPH expression in the dorsal raphe nuclei

**Figure 2.**
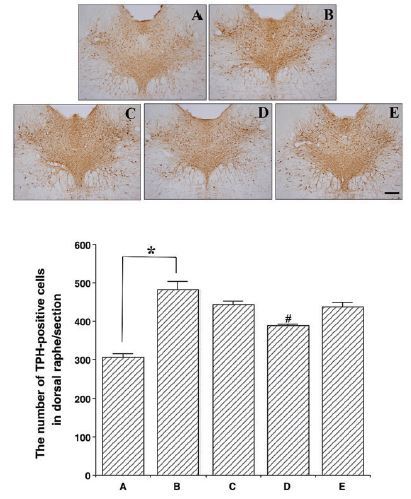
Effect colostrum serum on TPH expression in dorsal raphe. Upper: Photomicrograph of TPH-positive cells. The scale bar represents 250 μm (x4). Lower: number of TPH-positive cells in each group. A: control group B: exercise group, C: exercise and 50mg/kg of colostrum serum treatment, D: exercise and 100mg/kg of colostrum serum treatment, E: exercise and 200mg/kg of colostrum serum treatment. The data are presented as the mean ± standard error of the mean (S.E.M). * represents *p* < 0.05 compared to the control group. # represents *p* < 0.05 compared to the exercise

Photomicrographs of the TPH-positive cells in the dorsal raphe nuclei are presented in Fig. 2. The number of TPH-positive cells was 178.22 ± 12.15 for the control group, 359.11 ± 19.68 for the exercise group, 320.33 ± 18.93 for the exercise + 50 mg/kg colostrum serum group, 228.33 ± 11.06 for the exercise + 100 mg/kg colostrum serum group, and 304.33 ± 9.78 for the exercise + 200 mg/kg colostrum serum group. These results indicated that exhaustive exercise induces an overexpression of TPH in the dorsal raphe nuclei, and that treatment with colostrum serum alleviated the exhaustive exercise-induced expression of TPH in the dorsal raphe nuclei (F_(4,45)_=17.78, p<0.01). Specifically, treatment with 100 mg/kg of colostrum serum significantly suppressed TPH expression in the dorsal raphe nuclei when compared to the exercise group (p<0.01). 

### Effects of colostrum serum on 5-HT expression in the dorsal raphe nuclei

**Figure 3.**
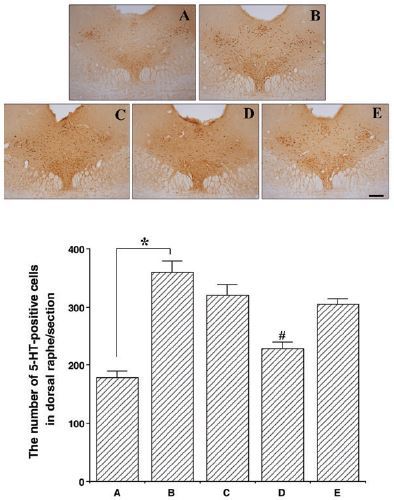
Effect colostrum serum on 5-HT expression in dorsal raphe. Upper: Photomicrograph of 5-HT-positive cells. The scale bar represents 250 μm (x4). Lower: number of 5-HT-positive cells in each group. A: control group B: exercise group, C: exercise and 50mg/kg of colostrum serum treatment, D: exercise and 100mg/kg of colostrum serum treatment, E: exercise and 200mg/kg of colostrum serum treatment. The data are presented as the mean ± standard error of the mean (S.E.M). * represents *p* < 0.05 compared to the control group. # represents *p* < 0.05 compared to the exercise group.

Photomicrographs of 5-HT-positive cells in the dorsal raphe nuclei are presented in Fig. 3. The number of 5-HT-positive cells was 307.11 ± 9.25 in the control group, 483.00 ± 20.32 in the exercise group, 444.22 ± 9.99 in the exercise + 50 mg/kg colostrum serum group, 388.22 ± 4.36 in the exercise + 100 mg/kg colostrum serum group, and 438.00 ± 11.46 in the exercise + 200 mg/kg colostrum serum group. These results indicated that exhaustive exercise induced an overexpression of 5-HT in the dorsal raphe nuclei, and that treatment with colostrum serum alleviated the expression of 5-HT in the dorsal raphe nuclei induced by exhaustive exercise (F_(4,45)_=15.68, p<0.01). Specifically, treatment with 100 mg/kg of colostrum serum significantly suppressed 5-HT expression in the dorsal raphe nuclei when compared to the exercise group (p<0.001). 

### Effects of colostrum serum on 5-HT_1A_ and 5-HTT expression in the dorsal raphe nuclei

**Figure 4.**
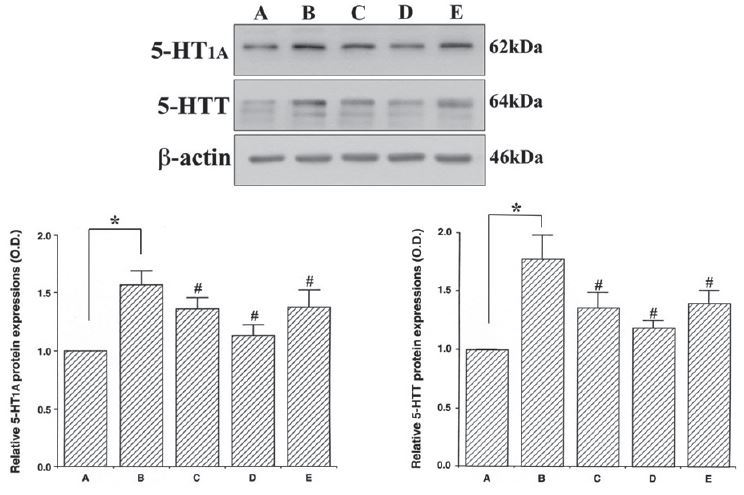
Effect colostrum serum on 5-HT_1A_ and 5-HTT expression in dorsal raphe. A: control group B: exercise group, C: exercise and 50mg/kg of colostrum serum treatment, D: exercise and 100mg/kg of colostrum serum treatment, E: exercise and 200mg/kg of colostrum serum treatment. The data are presented as the mean ± standard error of the mean (S.E.M). * represents p < 0.05 compared to the control group. # represents p < 0.05 compared to the exercise group.

The 5-HT_1A_ and 5-HTT protein levels are shown in Fig. 4. When the level of 5-HT_1A_ in the control group was set at 1.00, the level of 5-HT_1A_ was 1.57 ± 0.12 in the exercise group, 1.36 ± 0.10 in the exercise + 50 mg/kg colostrum serum group, 1.13 ± 0.09 in the exercise + 100 mg/kg colostrum serum group, and 1.37 ± 0.15 in the exercise + 200 mg/kg colostrum serum group (F_(4,45)_=8.48, p<0.01). When the level of 5-HTT in the control group was set at 1.00, the level of 5-HTT was 1.78 ± 0.20 in the exercise group, 1.36 ± 0.13 in the exercise + 50 mg/kg colostrum serum group, 1.19 ± 0.06 in the 100 mg/kg colostrum serum group, and 1.40 ± 0.11 in the 200 mg/kg colostrum serum group (F_(4,45)_=25.78, p<0.001). These results indicated that exhaustive exercise induces an overexpression of 5-HT_1A_ and 5-HTT in the dorsal raphe nuclei, and that treatment with colostrum serum suppressed the exhaustive exercise-induced expression of 5-HT_1A_ and 5-HTT in the dorsal raphe nuclei. All doses of colostrum serum significantly suppressed 5-HT_1A_ and 5-HTT expression (p=0.012, p<0.001 respectively), with 100 mg/kg being the most effective dose. 

## DISCUSSION

The fatigue that accompanies exercise is thought to originate from metabolic changes in muscle, such as muscle exhaustion and modifications of the CNS resulting in a decrease in motor neuron impulses to muscles^8, 23, 24^. The increased brain TPH concentrations and 5-HT synthesis induced by exercise may promote central fatigue and suboptimal exercise performance ^24^. 

Serotonin is mainly synthesized from serotonergic neurons in the raphe nuclei, which regulate the release of serotonin throughout the brain, and from neurosecretory cells in the pineal gland^25-27^. TPH is a crucial enzyme for the regulation of both serotonin synthesis and levels. The serotonin transporters (5-HTT) and 5-HT_1A_/5-HT1B autoreceptors also play important roles in the regulation of extracellular levels of serotonin in the raphe nuclei. Increased TPH activity during prolonged exercise increases the activity of serotonin, and this situation may cause a loss of central motivation and drive, and increase the lethargy that underlies fatigue^28^. Previous studies have shown that excessive exercise can lead to increased serotonin synthesis and TPH expression in the dorsal raphe nuclei, and this was linked to exhaustion during running^7, 29^.

Our study found that endurance exercise performance was effectively enhanced in the group that received colostrum serum compared to the control group. Furthermore, in addition to the enhancement of exercise performance, the expressions of 5-HT, TPH, 5-HT_1A_, and 5-HTT were suppressed in the dorsal raphe nuclei of the animals that received colostrum serum. These results suggest that colostrum serum (100 mg/kg) increased exercise performance and inhibited the serotonergic system in the dorsal raphe nuclei. 

Colostrum intake for six weeks has been shown to enhance immunity, reduce the concentration of creatine kinase (an indicator of muscle damage), and reduce the concentration of immunoglobulin G (IgG), while also increasing the concentration of cortisol. These effects improve the resistance against stress in marathon athletes^30^. Moreover, colostrum intake for nine weeks has been shown to increase the resilience of elite female rowing athletes^31^. Furthermore, a low dose of colostrum enhanced the athletic performance of cycling athletes during high-intensity training^32^. However, in a study involving two separate tests of physical performance in exercises of gradually increasing intensity to reach exhaustion separated by a 20 min partial recovery, colostrum intake for eight weeks did not induce performance differences during the first trial. However, after 20 min of passive recovery, exercise performance was shown to be increased by 5.2% on average during the second trial^33^. These results suggest a potential role for colostrum in increasing resilience and the resistance against fatigue. The immunological factors and bioactive substances that are present in colostrum are mainly constituents of colostrum serum. These include immune system molecules, growth factors, antibodies, vitamins, minerals, enzymes, and amino acids^34^. The major bioactive components of colostrum include IgG, tumor necrosis factor (TNF), interleukin-1,2,6 (IL-1,2,6), lactoferrin (LF), transforming growth factor (TGF), insulin-like growth factor (IGF-1,2), epidermal growth factor (EGF), and platelet-derived growth factor (PDGF)^35-37^. The molecular weight of these bioactive molecules ranges from 5–10 kDa, and colostrum contains 10 to 500 times more of these bioactive substances than normal milk. 

## CONCLUSIONS

Our study showed that moderate intake of colostrum serum enhanced exercise performance and suppressed the serotonergic system in the rat dorsal raphe nuclei. These results suggest that the intake of colostrum serum effectively suppressed the central fatigue induced by exhaustive exercise, and possibly alleviated the disruption to the immune system through the suppression of the central serotonergic system. Overall, our study suggests that colostrum serum has beneficial effects on the central fatigue induced by intensity exercise, which may have implications for social, community, and human life. 
